# Is oxidative stress - antioxidants imbalance the physiopathogenic core in pediatric obesity?

**DOI:** 10.3389/fimmu.2024.1394869

**Published:** 2024-08-08

**Authors:** Ancuta Lupu, Silvia Fotea, Elena Jechel, Iuliana Magdalena Starcea, Ileana Ioniuc, Anton Knieling, Delia Lidia Salaru, Maria Oana Sasaran, Olga Cirstea, Ninel Revenco, Cristina Maria Mihai, Vasile Valeriu Lupu, Alin Horatiu Nedelcu

**Affiliations:** ^1^ Pediatrics, “Grigore T. Popa” University of Medicine and Pharmacy, Iasi, Romania; ^2^ Clinical Medical Department, Faculty of Medicine and Pharmacy, “Dunarea de Jos” University, Galati, Romania; ^3^ Faculty of Medicine, “Grigore T. Popa” University of Medicine and Pharmacy, Iasi, Romania; ^4^ Pediatrics, “George Emil Palade” University of Medicine, Pharmacy, Science and Technology, Targu Mures, Romania; ^5^ Pediatrics, Nicolae Testemitanu State University of Medicine and Pharmacy, Chisinau, Moldova; ^6^ Pediatrics, Faculty of Medicine, “Ovidius” University, Constanta, Romania

**Keywords:** oxidative stress, endogenous antioxidant systems, exogenous antioxidants, obesity, diet, immunity, child

## Abstract

Despite the early recognition of obesity as an epidemic with global implications, research on its pathogenesis and therapeutic approach is still on the rise. The literature of the 21st century records an excess weight found in up to 1/3 of children. Both the determining factors and its systemic effects are multiple and variable. Regarding its involvement in the potentiation of cardio-vascular, pulmonary, digestive, metabolic, neuro-psychic or even dermatological diseases, the information is already broadly outlined. The connection between the underlying disease and the associated comorbidities seems to be partially attributable to oxidative stress. In addition to these, and in the light of the recent COVID-19 pandemic, the role played by oxidative stress in the induction, maintenance and potentiation of chronic inflammation among overweight children and adolescents becomes a topic of interest again. Thus, this review’s purpose is to update general data on obesity, with an emphasis on the physiopathological mechanisms that underlie it and involve oxidative stress. At the same time, we briefly present the latest principles of pathology diagnosis and management. Among these, we will mainly emphasize the impact played by endogenous and exogenous antioxidants in the evolutionary course of pediatric obesity. In order to achieve our objectives, we will refer to the most recent studies published in the specialized literature.

## 1 Introduction

Being defined as an increase in the body mass index (BMI) above the 95th percentile/at +1-2 standard deviations compared to the reference average for age and sex, overweight or obesity is a chronic condition with multisystemic effects. Thus, it is frequently associated with hypertension, metabolic disorders (e.g., dyslipidemia, prediabetes or type 2 diabetes), neuro-psychological, gastroenterological (e.g., non-alcoholic fatty liver, gallstones, gastroesophageal reflux), renal, respiratory, orthopedic damage or dermatological (e.g., atopic dermatitis, acanthosis nigricans) (1–6). Also, a positive correlation was also identified with regard to sleep apnea or the increase in the flu severity score among children with obesity (7, 8).

Recent estimates according to the World Obesity Atlas 2023 place pediatric obesity as a chronic condition that affects 2.6 million children under the age of 5 and up to 175 million children between the ages of 5 and 19. The gender ratio in the second category is against girls (103 million cases) (9). Regarding the division by sex, it is certified that girls/women present an increased risk compared to boys partly due to hormonal variability (10). The highest incidence is noted in Western countries, while in low-income countries or in countries with a low development index, pediatric obesity is better represented among families with above-average income (11). An additive risk factor is identified among “medically complex” children, namely those children with chronic health conditions, significant functional limitations and increased risk of hospitalization (12). The prevalence of childhood obesity has grown rapidly in the last 40 years, doubling. According to current trends, it is predicted that in approximately 30 years, up to 25% of children will suffer from excessive weight gain (10, 13). In addition, the literature attests to the direct risk of developing obesity in adult life of the population with excess weight in childhood (14). In this situation, the pediatrician has a key role in reducing the global effects of obesity. Due to the nature of the population, he cares for, knowing and countering the pathological processes that disrupt the homeostasis of the internal environment in obesity and the associated comorbidities will lead, in the long term, to reducing the burden on adults and the medical system (15). To facilitate inclusion in risk groups and reduce childhood obesity, Sonoda R. et al. imagined a prediction model based on seven binary variables (16).

The defining imbalance of obesity is represented by a high caloric intake, doubled by a low consumption. Their main regulators are metabolic rate, appetite, eating habits and lifestyle dynamics. Genetic factors and individual characteristics (e.g., birth weight, breastfeeding period) may predispose to obesity, although a more important imprint is attributed to the environment. Therefore, the increase in the incidence of excess weight in recent years can be more correctly attributed to environmental changes, educational deficiencies or western-type diets. In this direction, current research attests to the involvement of numerous biomarkers (microRNA, adipocyte balance, oxidative stress, blood cell profile, nutrients and microbiota) determinants of the development of excess weight and systemic damage (2, 13, 17–19). Therefore, in addition to the disturbances stated above, excessive weight gain together with the accompanying chronic inflammation represents a continuous source of oxidative stress. The latter seems to be the crossroads in triggering the comorbidities that accompany obesity. Therefore, stimulating interest in research and countering it can be crucial initiatives, additive to new prevention strategies, to improve quality of life and ameliorate systemic decline (20, 21).

Therefore, knowing the global impact of obesity and its comorbidities, both in pediatric and adult age, we tried to broaden the horizons in terms of understanding the physiopathological mechanisms underlying the disturbances and the means by which they can be counteract. In this sense, we chose as a topic of interest the implications of oxidative stress in excessive weight gain. The antioxidant substances are multiple, they can be briefly distinguished into endogenous and exogenous. Although their importance is similar, in the management of the overweight patient, counteracting the main nutritional deficits is essential, both for the restoration of adequate levels, and from the perspective of the function of cofactors for the endogenous substances played by some of them. We caried out a narrative review of the scientific literature from the last decades, by accessing international databases (PubMed, Google Scholar, Web of Science, Scopus and Embase). To facilitate the search, we used terms such as “obesity”, “oxidative stress”, “antioxidant enzymes”, “exogenous antioxidants”, “antioxidant vitamins”, “antioxidant trace minerals” or “oxidative stress modulators”.

## 2 The impact of oxidative stress in the dynamics of obesity

Obesity has multiple causal determinations. Among these we note the environmental factors (stress, drugs, surgical interventions, chemical substances, diet, sleep schedule, physical activity), genetic predisposition, as well as the disruption of the homeostasis of the internal environment, objectified by oxidative stress reactions. Therefore, maintaining the redox balance in the adipose tissue is an important objective due to its implications in the decrease of the organic antioxidant capacity, the formation of free radicals and reactive species and in the pathogenesis of the metabolic syndrome associated with obesity (22–24).

Oxidative stress is defined as the disturbance of the balance between the production of oxidizing agents and the antioxidant defense, materialized by the formation of free radicals. Biochemical substrates that interfere with its production include the mitochondrial respiratory chain and enzymes (e.g., nicotinamide adenine dinucleotide phosphate oxidase, xanthine oxidase, lipoxygenases, cyclooxygenases, cytochrome P450 enzymes, or uncoupled nitric oxide synthases). This results in reactive species derived from oxygen and nitrogen (superoxide anion radicals, hydroxyl, alkoxyl, lipidic peroxyl, nitric oxide and peroxynitrite). It imprints a state of suffering on the body, sensitizing it. There is an alteration of the intra- and extra-cellular components. It is objectified by structural and functional imbalances of nucleic acids, proteins or lipids. Among its consequences, we note the over-expression of oncogenic genes, the generation of mutagenic compounds, the promotion of atherogenic activity or inflammation. Current research incriminates the existence of oxidative stress as a physiopathogenic mechanism in a variety of pathologies, depending on genetic susceptibility. Among these we highlight hypertension, atherosclerosis, obstructive pulmonary disease, diabetes, osteoporosis, cancer or even infertility (25–31).

The main method to combat the negative effects of oxidative stress is the detoxification of metabolic byproducts. This is done by enzymatic or non-enzymatic components. The first category includes superoxide dismutase (SOD), glutathione peroxidase (GPx), glutathione reductase, glutathione S-transferase, catalase (CAT), thioredoxin reductase, peroxiredoxins (Prx), ubiquinone oxidoreductase and heme oxygenase-1 (HO-1). Recently introduced in research, we find the paraoxonase family (PON) or aryl dialkyl phosphatases, enzymes strongly correlated with the accompanying pathologies of obesity (32). Another important determinant is nuclear factor erythroid 2 (Nrf2), defined as a regulator of cellular resistance to oxidants. Its action is manifested on a wide range of genes with implications in antioxidant modulation, immune or inflammatory responses, tissue remodeling, carcinogenesis and cognitive balance. Knowing and understanding its roles can lead to the explanation of the mechanisms underlying the correlation between oxidative stress and induced pathologies. At the same time, it lays the foundations for new therapeutic perspectives (33, 34). Melatonin is another magical compound, with functions in regulating the circadian rhythm, sleep, inhibiting cancer, but also detoxifying free radicals (35). Dietary antioxidant substances (non-enzymatic) such as vitamin A, C, E and other plant compounds (flavonoids, tannins, lignins) are added to all of these. Likewise, the trace elements (zinc, manganese, selenium) should not be lost sight of, substances assimilated as enzyme regulators (32).

**Figure 1:**
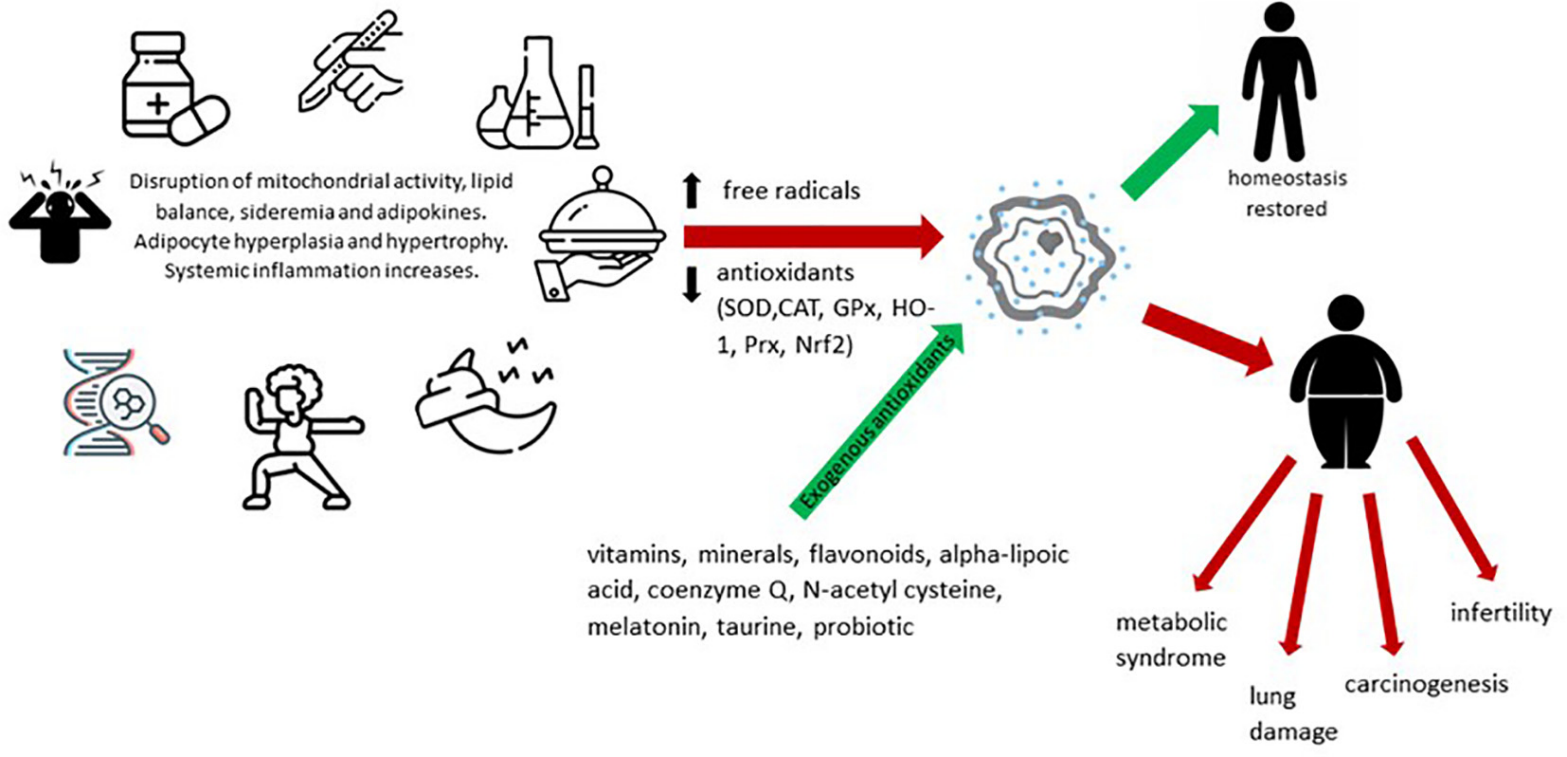
Dynamics of oxidative stress and its implications in pathogenesis of obesity.

Oxidative stress in obesity has been shown to be directly correlated with fat mass. Precipitating factors in this case include altered nutritional balance, hyperglycemia, hyperlipidemia and chronic inflammation. Also, the high-carbohydrate diet potentiates oxidative stress, an interaction objectified by increasing lipid peroxidation and protein carbonylation, doubled by the reduction of glutathione and antioxidant levels. Other classical risk factors incriminated are pollution, radiation, pesticides or other toxic substances, infections and surgical interventions. The consequences are diverse, among which we mention the influence of myocardial contractility, vascular remodeling, insulin resistance or adiponectin secretion. This explains the correlation between obesity, inflammation and various chronic pathologies that make up the metabolic syndrome (diabetes, heart disease) or not (carcinogenesis, asthma, COVID-19) (36–39). Summarizing the above, **Figure 1** shows the enzymatic and non-enzymatic balance involved in the pathogenesis of oxidative stress in obesity. Imbalances at this level interfere, in addition to inflammation, with mitochondrial activity, adipogenesis, lipolysis and lipogenesis, appetite and iron metabolism.

### 2.1 Mitochondrial activity

**Figure 2:**
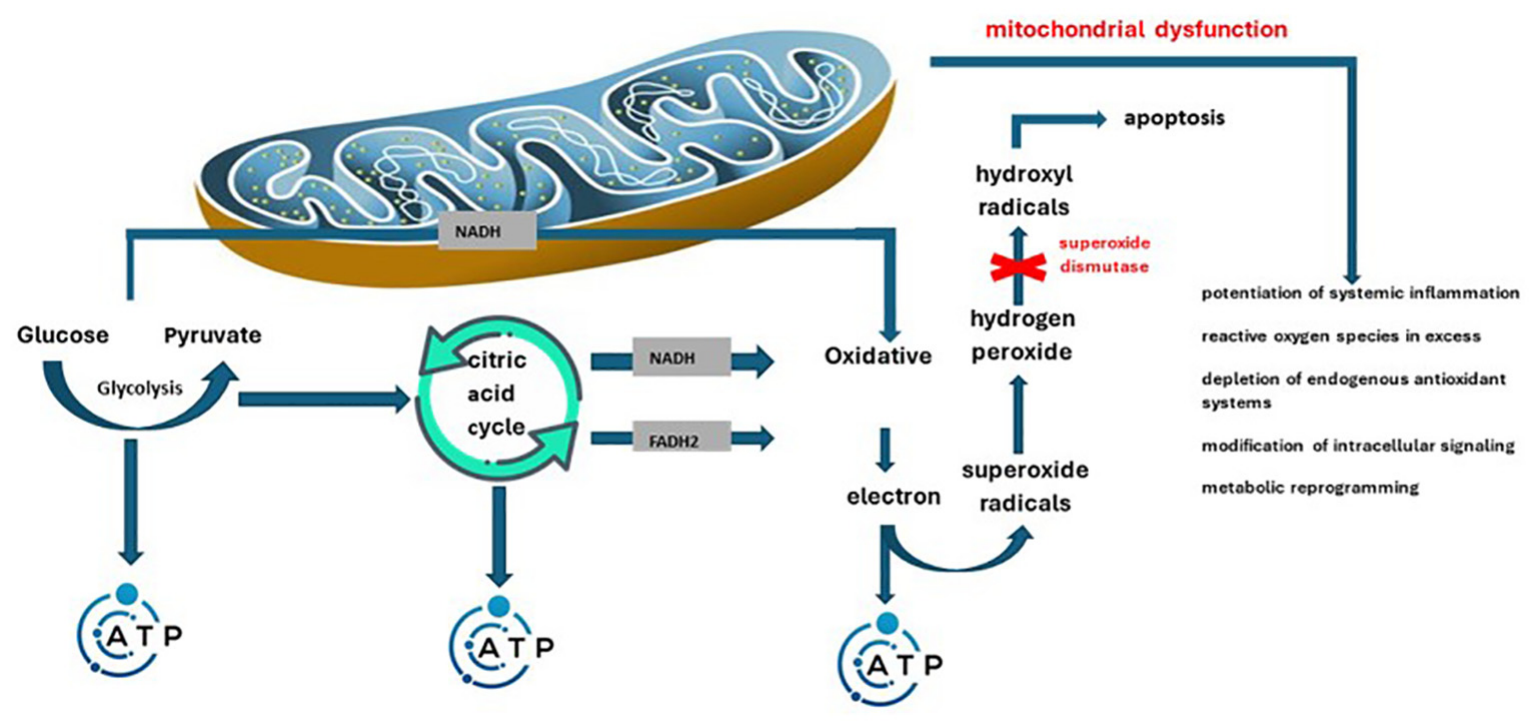
Pathological mechanisms through which mitochondrial dysfunction intervenes in the potentiation of the oxidative status and systemic damage.

The main roles of mitochondria are represented by production of energy (adenosine triphosphate – ATP) depending on cellular needs and fighting infections by promoting reactive oxygen species (40). In this sense, mitochondrial/peroxisomal oxidation of fatty acids, as well as excessive oxygen consumption can produce reactive oxygen species (41). Mainly, during the conversion to ATP, a small amount of high-energy electrons may deviate from the programmed path, thus generating superoxide radicals. They are enzymatically or spontaneously transformed into hydrogen peroxide and later into hydroxyl radicals, dangerous molecules (they can indicate cell apoptosis). The effective antioxidant mechanism in this sense is superoxide dismutase. The enzyme switches the process towards the formation of hydrogen peroxide. Later glutathione peroxidase will determine its transformation into water (42). Inflammation together with the overproduction of free radicals in obesity predispose to the appearance and maintenance of mitochondrial dysfunction. This is defined as decreased biogenesis, altered membrane potential, altered mitochondrial gene expression, and reduced ATP production (43). Another variable encountered in this case is the excess supply of nutrients. The consequence is the overwhelm of the Krebs cycle and the mitochondrial respiratory chain. This is how mitochondrial dysfunction is precipitated, which results in the formation of an increased amount of reactive oxygen species. The two mechanisms therefore intertwine, aggravating in parallel both mitochondrial dysfunction, as well as inflammation and increased insulin resistance specific to obesity (40, 42). The exposed changes, related to mitochondrial biogenesis, enzyme balance and the consequences of the two, were also reported by Zamora-Mendoza R. et al. (44). Summarizing the above, **Figure 2** illustrates the way in which mitochondrial dysfunction induces an increase in oxidative status and disruption of the homeostasis of the internal environment.

### 2.2 Adipogenesis

**Figure 3:**
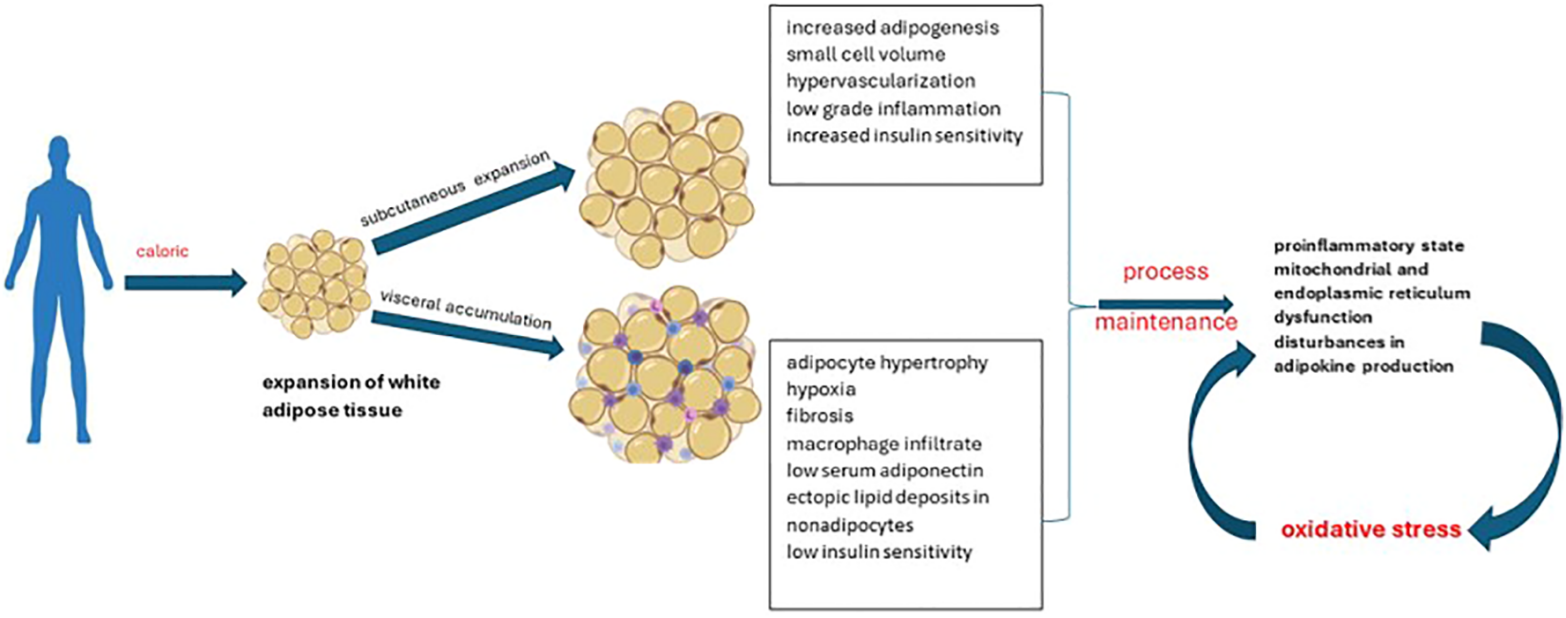
Patterns of obesity depending on the expansion of white adipose tissue.

Adipose tissue normally represents up to 30% of body mass. Its roles in the body are variable, being briefly divided into structural (support) and functional. Depending on its distribution, it can be classified into subcutaneous adipose tissue and visceral adipose tissue. Its structural and functional characteristics facilitate its division into 3 categories: white, brown or beige adipose tissue. Functionally, the main role of white adipocytes is to store energy, while brown adipocytes intervene in thermogenesis. Thermogenesis occurs by stimulating uncoupling proteins, the result being the conversion to produce heat instead of ATP. Beige adipocytes share similar characteristics to brown ones, but being distributed in white adipose tissue deposits (45). Brown adipose tissue seems to have a metabolic activity directly correlated with the volume of muscle mass; an aspect partially explained by the presence of common precursors with muscle cells (myf5-positive precursors similar to myoblasts). Also, it is better represented in the pediatric population, unlike adults. The investigation recommended for its evaluation is positron emission tomography, although the results may be influenced by age, degree of sexual development, medication used, fat accumulation, disease state, plasma glucose concentration, radiotracer dose, season or temperature during examinations. Antioxidant supplementation has beneficial effects on the dynamics of brown adipose tissue (45–47). Current data from the literature attests to the discrepancy between white adipose tissue/brown adipose tissue in the pathogenesis of obesity. If the excessive distribution of the first one is found in the case of overweight people, the brown tissue protects against weight gain and its comorbidities (insulin resistance, dyslipidemia). However, the way of distribution of white adipose tissue should not be neglected. It differentiates pathological obesity from “metabolically healthy” obesity (**Figure 3**). The second case is characterized by the expansion of subcutaneous deposits, adipocyte hyperplasia and limited ectopic lipid deposition (48, 49).

The imbalance in adipose tissue, characteristic of obesity (hypertrophy, hyperplasia), is accompanied by a pro-inflammatory state, mitochondrial and endoplasmic reticulum dysfunction and, consequently, increased oxidative stress at this level. This in turn induces disturbances in the production of adipokines. The link between the two is strengthened by the direct correlation between oxidative damage markers and BMI, body fat percentage, low-density lipoprotein (LDL) oxidation, and triglyceride (TG) levels. The consequences are reflected in the increased risk of developing comorbidities in the medium and long term (50, 51). In agreement with the functional characteristics of adipose tissue, studies on adults attest to lower levels of oxidative stress in men, compared to women. At the same time, the correlation with environmental factors is certified (e.g., the use of oral contraceptives, hormonal therapies) (52). In turn, oxidative stress promotes inflammation by activating the components of innate immunity and modulating the activation of the nucleotide oligomerization domain protein-1 (NOD1). Following the activation of NOD1, there is an increase in NADPH oxidase (NOX) 1 and 4. From a therapeutic point of view, it seems that the manipulation in the descending direction of NOX1/4 decreases oxygen free radicals, in parallel with the increase in catalase-type antioxidant enzymes and SOD (53, 54).

### 2.3 Lipogenesis and lipolysis

**Figure 4:**
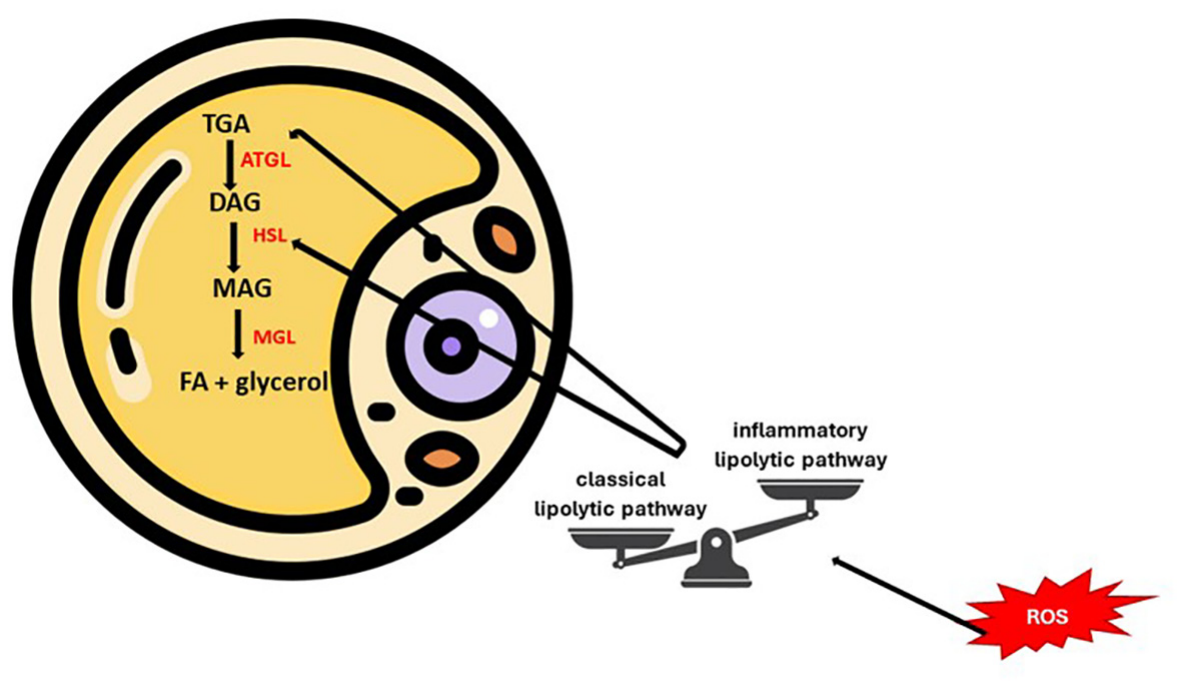
Key points of lipolytic balance modulated by oxidative status.

Lipids fulfill important biological roles such as energy source, structural components or signaling mediators. The tissues involved in lipogenesis are the liver and adipose tissue (white/brown). This modulates, in addition to systemic energy homeostasis, the function of the immune and nervous systems. Thus, lipid metabolism disturbances can be accompanied by pathological consequences, partly due to membrane remodeling (55–57). The metabolic integrity of the body can be estimated by evaluating the ability of adipocytes to convert glucose into lipids (*de novo* lipogenesis), a process affected in the case of insulin resistance (58). Therefore, in obesity, the classic lipolytic pathway is switched to an alternative, inflammatory pathway. Reactive oxygen and nitrogen species can influence these pathways at different levels, with consequences dependent on concentration, reactivity and source (**Figure 4**). Mainly excessive and prolonged lipolysis (lipotoxicity) causes structural and functional changes in adipose tissue. The physiopathogenic cascade ends with the impairment of insulin sensitivity, maintaining the vicious circle of lipolysis. In conclusion, we emphasize the importance of antioxidants in restoring the homeostasis of the internal environment (59, 60).

### 2.4 Leptin – adiponectin balance

**Figure 5:**
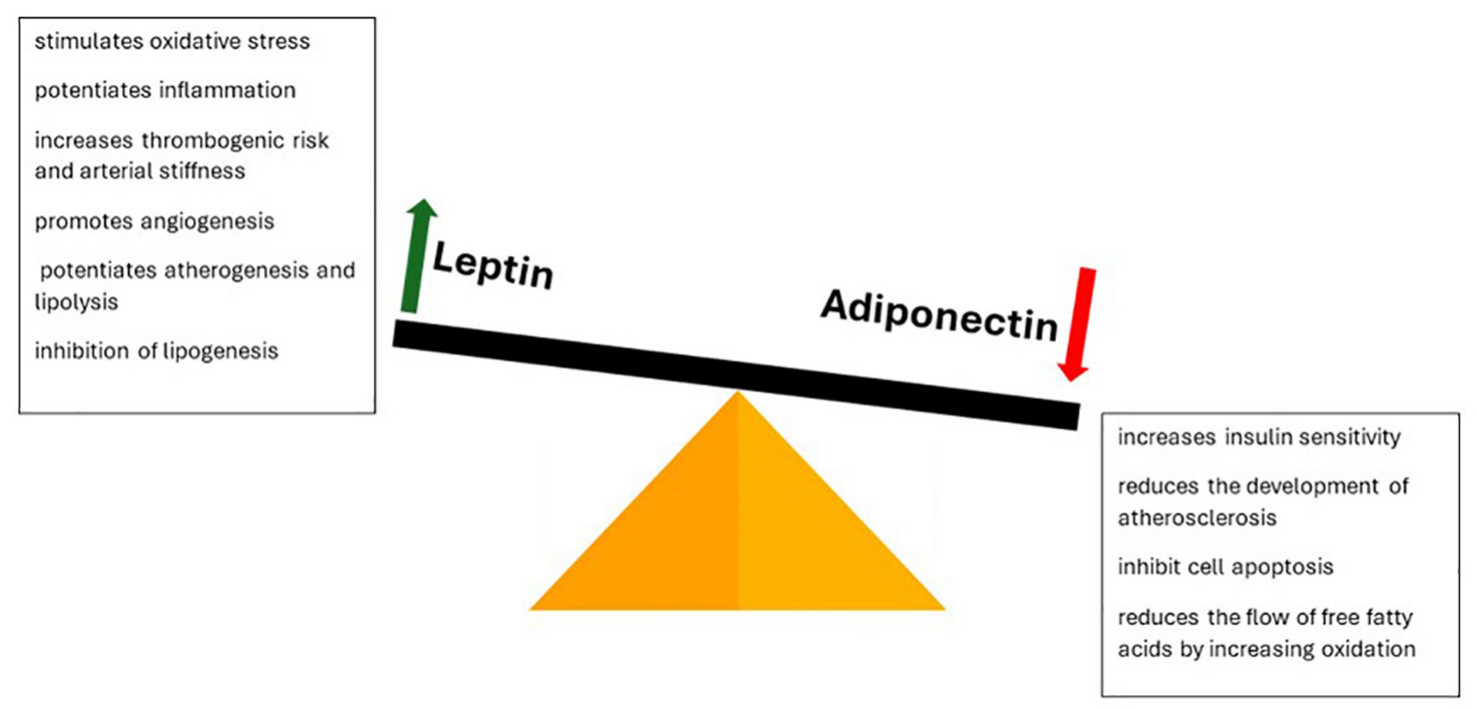
Leptin-adiponetic antithesis in dictating obesogenic risk and its complications.

Adipose tissue also fulfills an endocrine role, secreting a series of bioactive molecules (adipokines) that influence the internal balance. Their production is influenced by insulin, catecholamines and adiposity. By this we mark another stage of the physio-pathological process influenced by oxidative stress, namely the leptin/adiponectin balance. The functions performed by the two molecules are contradictory (**Figure 5**). Leptin is involved in appetite regulation by modulating dopamine secretion by the limbic system. At the same time, it stimulates oxidative stress, inflammation, thrombogenic risk, arterial stiffness, angiogenesis, atherogenesis and lipolysis, inhibiting lipogenesis. This explains the increased cardiovascular risk characteristic of obesity. At the opposite pole, adiponectin is an anti-inflammatory molecule that increases sensitivity to insulin, reduces the development of atherosclerosis, cell apoptosis and the flow of free fatty acids, in parallel with the increase in their oxidation. Its action is mainly achieved through the interaction with ceramidase, the enzyme involved in the intracellular balance of ceramide. Oxidative stress suppresses its production, thus increasing the risk of associated comorbidities. Supplementation with antioxidants restores the circulatory balance and reduces morbidity. Other adipokines involved in the modulation of comorbidities are adipsin, resistin, visfatin, omentin and apelin (41, 61–64).

### 2.5 Iron metabolism

**Figure 6:**
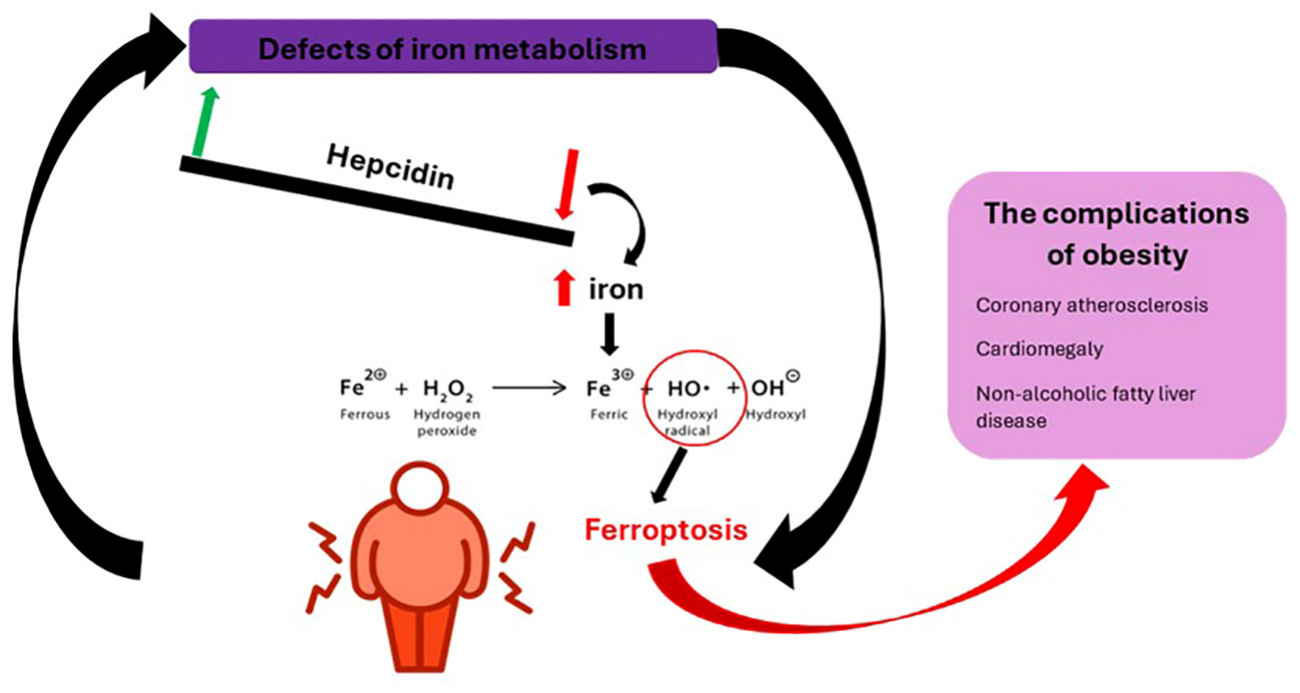
The physiopathogenic mechanism of obesity complications following iron metabolic defects.

Iron metabolism has a double value in obesity, disturbances at this level can be both a consequence of overweight and a cause. The common point between the two entities seems to be represented by macrophages. On the one hand, it can be influenced by chronic inflammation, its deficit affecting the function of hemoproteins and non-heme proteins. In this case, iron deficiency is accompanied by a low value of ferritin, in contrast to that of hepcidin, and a negative correlation between transferrin saturation and adiposity. On the other hand, excess iron can induce the formation of oxygen free radicals (hydroxyl radical) through the Fenton reaction whose course and consequences are illustrated in **Figure 6** (65, 66). Added to these are the increase in lipogenesis, the reduction of lipolysis and the promotion of mitochondrial dysfunction (67). This is how ferroptosis (regulated non-apoptotic cell death) occurs, characterized by lipid peroxidation when the endogenous antioxidant status of the cell is compromised. The remedies in this situation are represented by dietary restriction, the use of iron chelators, lipophilic antioxidants (vitamin E), ferostatin-1, liproxstatin-1 or polyphenols (68, 69).

### 2.6 Inflammation

Chronic, low-grade inflammation is the main mechanism incriminated in promoting obesity-induced damage. This is characterized by a continuous activation of the innate immune system (70). The cause is the accumulation of lipids in adipocytes, leading to adipocyte stress and local hypoxia following tissue hypertrophy and hyperplasia. Thus, necrosis and infiltration by macrophages occurs. There is an increase in the production of pro-inflammatory mediators (interleukin-6 and 1, tumor necrosis factor α, monocyte chemoattractant protein 1) and prothrombotic (plasminogen activator inhibitor-1). Additionally, deficiencies of the oxidizing and antioxidant system are noted. At the same time, the local inflammation spreads to the systemic level (42, 71, 72). Tumor necrosis factor α (TNF-α) has also been linked to endothelial dysfunction, increased atherogenic risk, development of insulin resistance and diabetes. This is achieved through four essential means, namely the activation of nuclear factor κB (NF-κB), increasing the release of free fatty acids in adipocytes, blocking the synthesis of adiponectin and modulating the phosphorylation of tyrosine residues, an essential substrate in intracellular signaling. Regarding IL-6, its levels are directly correlated with BMI, insulin resistance and carbohydrate intolerance. Besides these, it regulates the levels of adipokines (inhibits visfatin and adiponectin) (41).

In conclusion, we present the results obtained by Matusik P. et al. who reiterate, based on their research, the positive correlation between oxidative disturbances, sports, excess adipose tissue and its hormonal activity. However, he notes that in the case of patients practicing sports training (chronic physical activity), the antioxidant defense was more potent. In consensus, Huang CJ. et al. explains the connection between sport and the oxidant-antioxidant balance, emphasizing the dependence on the individual characteristics of the patient, the type of physical activity practiced, intensity and period. Also, a brief distinction is made of the mechanisms by which aerobic and anaerobic physical activity influence the internal balance. The final result is, in both situations, represented by the increase of stress markers (73, 74). Similar findings have been identified in the literature regarding non-alcoholic fatty liver disease, another component of the metabolic syndrome, frequently associated with obesity (75).

## 3 Antioxidant systems

Reactive oxygen species in excess can induce apoptosis, necrosis or autophagy. The main pathways targeted in this process are mTOR activity, adenosine monophosphate-activated protein kinase (AMPK), C-Jun-N-terminal kinase (JNK)/P53, and the balance between serine/threonine receptor-interacting protein kinase 3 (RIP3) and RIP1 (76). For these reasons, knowing and potentiating the main endogenous antioxidants is vital in the evolution of patients.

### 3.1 Enzymatic antioxidants

Superoxide dismutase and catalase are two key enzymes in returning the body to its equilibrium state. The two participate in the elimination of toxic radicals through the initial conversion to hydrogen peroxide (H_2_O_2_) by SOD, which will then be degraded by catalase into oxygen and water. Thus, to estimate the level of expression of the two in the peripheral sagin cells, Mohseni R. et al. they turned to real-time polymerase chain reaction (PCR). The results confirmed the low levels among obese children, inversely correlated with BMI, fasting blood glucose, insulin resistance, LDL-Cholesterol, TG and systolic blood pressure (77). SOD is divided into 3 isoforms in mammals. These are the cytosolic copper-zinc dimeric form (SOD1), mitochondrial manganese tetrameric SOD (SOD2), and extracellular Cu/Zn tetrameric SOD (SOD3). Among them Erdeve O. et al. demonstrates the involvement of SOD3 in the antioxidant response since childhood (78, 79). At the same time, Özgen İT. et al. underlines the role of genetic polymorphisms (respectively VV alleles) of SOD2 Ala16Val in dictating the risk of developing oxidative stress associated with increased HOMA-IR score (80). In turn, CAT is a peroxisomal antioxidant enzyme, crucial in adipogenesis. Studies on erythrocytes report the possible correlation of single nucleotide polymorphisms (promoter variant -844A/G) and post-translational modifications of CAT with the risk of developing obesity and its comorbidities. At the same time, the low levels of CAT observed in obesity are partially due to increased S-nitrosation of the enzyme (81–83). Paradoxically, it remains contradictory if CAT overexpression in adipose tissue offers any benefit in terms of improving the metabolic profile or the balance of adipogenesis (84).

The main disadvantage of antioxidant enzymes is the inability of exogenous variants to provide benefits to damaged tissues. The main cause is the low permeability of the membrane. Thus, in order to achieve results, recombinant formulas are used, made up of SOD and cell-penetrating proteins (SOD-CPP). This formula can even cross the blood-brain barrier, reducing the expression of inflammatory factors and inhibiting NF-κB signaling pathways (85). Another form used is the SOD mimetic manganese metalloporphyrin (86).

Glutathione peroxidase is an antioxidant enzyme found at the cytoplasmic and mitochondrial level in mammals. The entire superfamily brings together eight isoforms, of which types 1-4 and 6 are selenoproteins, the rest based on cysteine. In humans, the most common isoform involved in oxidative balance is GPx1 (87). As a means of action, it maintains the balance between the necessary/damaging level of cellular oxidants by reducing hydrogen peroxide and soluble lipid hydroperoxides. The source of reducing equivalents used is glutathione. Enzyme balance is important because low levels of hydrogen peroxide are required for physiological processes such as growth factor-mediated signaling, formation of protein-disulfide bonds, and regulation of normal mitochondrial function. At the same time, a high level of GPx1 can induce dysregulation of islet insulin production and secretion, culminating in diabetes-like phenotypes. The repercussions at the pancreatic level consist of depletion of murine regenerating islet-derived protein 2 (REG2). Thus, maintaining optimal levels of GPx1 is crucial in modulating systemic inflammation and preventing associated metabolic, neurological, cardio-vascular and oncological risks (88–91). In this process, an important role is attributed to glutathione reductase. The enzyme is the main supplier of reduced glutathione, a necessary substrate for the proper development of the redox process (92). Independent of other variables that can influence the redox balance, it seems that seasonal factors (e.g., vitamin D) can modulate the reactions based on glutathione (93). In this sense, vitamin D supplementation showed benefits in increasing glutathione, GPx1 and SOD, reducing oxidative stress and suppressing ferroptosis (by activating the Nrf2 signaling pathway). In parallel, the neuroprotective effect exerted following the hypoxic-ischemic stimulus is noted (94).

Peroxiredoxins represent an enzyme superfamily, dependent on cysteine. Prx reduce over 90% of cellular peroxides. These have six subfamilies in their composition (95). They demonstrated their role in regulating adipogenesis and the metabolic/inflammatory implications of obesity. Among these we note diabetes, atherosclerosis, liver, cardiac or even reproductive damage (spermatogenesis) (96–100). Similar implications are noted in the case of paraoxodaxa-1, the thioredoxin/thioredoxin reductase system (Trx/TrxR), adipokines or heme oxygenase-1 (101–105).

### 3.2 Non-enzymatic antioxidants

Among the non-enzymatic molecules involved in the clearance of reactive oxygen species and the reduction of their harmful effects, we find vitamins (A, E and C) and other plant compounds (flavonoids, tannins, lignins, carotene, alpha-lipoic acid), chemicals such as coenzyme Q/ubiquinone (MitoQ), N-acetylcysteine (NAC) or trace elements (zinc, manganese, selenium). Compounds with special properties such as melatonin and taurine are added to these (32, 43, 76).

#### 3.2.1 Vitamins

Fat-soluble vitamins (A and E) are dietary constituents whose intestinal passage (absorption/elimination) occurs passively, without energy consumption. Vitamin A can be found as two forms, preformed (all-trans-retinol and its esters) and provitamin A (β-carotene). Of these, β-carotene, similar to other dietary carotenoid products, acts as an antioxidant substrate (106). Similarly, the distribution of vitamin E brings together 8 isoforms (four tocopherols and four tocotrienols - α -, β-, γ-). Vitamin E is ubiquitous in the body’s sites, although it lacks the possibility of endogenous synthesis. The functions performed by it spread over the allergic, inflammatory, atherogenic, metabolic (glycemic, lipidic), oncological balance, being also an important pillar in cardioprotection and neuroprotection. It is believed that the antioxidant activity of vitamin E is dependent on the number of methyl groups on the chroman ring. The antioxidant power includes α, β, γ and δ isoforms in descending order, while tocopherol is stronger than tocotrienol (107).

Based on previous findings, Guerendiain M. et al. certifies the involvement of the correlation between optimal levels of fat-soluble vitamins and reduced adiposity, greater weight loss and improved cardio-metabolic profile (108). In addition, Gama Oliveira MN. et al. include in the discussion another predisposing variable, namely the Q223R leptin receptor polymorphism (109). Another concern brought into discussion by Gajewska J. et al. is the risk of hypovitaminosis E or A in obese children, dependent on the BMI curve for weight loss through lifestyle interventions (110).

Vitamin C (ascorbic acid) fulfills in the body both a role in the antioxidant and immune balance (innate or adaptive) and as an enzyme cofactor. Through its functions, it promotes the integrity of the skin barrier, increases chemotaxis, phagocytosis, the generation of reactive oxygen species and microbial killing, reduces necrosis and post-infectious tissue damage and promotes the differentiation and proliferation of T and B lymphocytes. Thus, in inflammations and infections, subject to important reactive oxygen species, vitamin C has a double valence. While its deficiency induces the disruption of immune defense mechanisms, the infections themselves promote the deficiency due to inflammation and nutritional requirements, thus maintaining the vicious circle (111, 112). Current literature attests to the benefit of ascorbic acid in oncology, cardiovascular (hypertension, stroke, atherosclerosis) and nutritional (obesity) conditions. However, we emphasize the existence of gender influence, marked by a negative correlation between vitamin C intake and abdominal obesity in women. The mechanisms probably involved in this are represented by the modulation of adipocyte lipolysis, adrenal glucocorticoid levels, glucose metabolism (improves blood sugar and decreases glycosylation) and leptin secretion and reduces the inflammatory response (113–115). It is known that the optimal intake for a person of approximately 60 kg is 110 mg/day. Carr AC. et al. note that, to avoid vitamin C deficiencies, it is necessary to supplement with 10 mg/day for every 10 kg of excess weight (116).

Comparing the three vitamins, Xie D. et al. notes an increased antioxidant power of vitamin A compared to vitamin C. At the same time, vitamin E is more potent than vitamin A, the combination of the two not registering additional antioxidant benefits (117).

Vitamin D (25-hydroxyvitamin D) is another fat-soluble vitamin with a strong role in homeostasis of the internal environment. The main functions are based on the inflammatory, oxidative and mitochondrial balance. Its balance is influenced by a multitude of physical and environmental factors. In obesity in particular, hypovitaminosis D seems to be due to insufficient food intake, reduced exposure to sunlight due to the inclination towards a sedentary lifestyle, decreased intestinal absorption, hydroxylation in adipose tissue or accumulation in fat. To these is added the genetic predisposition marked by the variation of the vitamin D receptor gene (118–120). Studies on murine models attest to the beneficial effects of vitamin D supplementation on oxidative stress and inflammatory markers in the overweight group (121). Similar findings were reported by Ionica M. et al., in the study on the adult, overweight and obese population. They also record the inverse correlation between the serum level of the vitamin and the amplitude of adipose oxidative stress (122). Continuing the reasoning, Usman M. et al. objectifies, by studying obese pediatric patients aged between 10-18 years, the positive link between adipose markers and oxidative DNA damage. In conclusion, the three entities (obesity, hypovitaminosis D and DNA damage) represent predictive markers of genomic instabilities (123). In conclusion, Filgueiras MS. et al. underlines the importance of screening for vitamin D status in light of the association with non-conventional cardiometabolic markers (C-reactive protein, IL-6, cathepsin S, vascular cell adhesion molecule-1, malondialdehyde, myeloperoxidase, 3-nitrotyrosine and SOD) in the pediatric population (124). It is estimated that increasing vitamin D by approximately 9 ng/ml would counterbalance this negative effect of visceral adiposity (125).

#### 3.2.2 Microelements

Microelements are important chemical structures in the balance of the internal environment. By their nature, they fulfill multiple functions, entering into the composition of enzymes, vitamins, hormones and pigments. By far the most important role is that of an enzymatic cofactor, modulating organic biochemical processes through their level. Currently, the medical literature attests to the presence of deficiency in trace elements in children and adolescents with obesity. Among these, the biggest shortage seems to be recorded in what concerns copper (126). Although the relationship between obesity and nutritional deficiencies remains open to research, Błoniarz J. et al. links the metabolism of carbohydrates, fats and implicitly the appearance of secondary obesity to the balance of chromium, zinc, copper, manganese, iron or nickel (127). In addition, Luque-Díaz MJ. et al. underlines the existence of hyperzincuria, hypocupruria, hypozincemia and hypercupremia in groups of obese patients, unlike controls (128). Not being the object of the current study, we choose to expose in what follows its implications from the perspective of oxidative stress.

We therefore consider zinc, manganese, selenium and magnesium as the main elements involved in antioxidant regulation. The function of zinc includes a wide range of biological processes such as cell proliferation, immune function and defense against free radicals (through the synthesis of metallothioneins - they reduce hydroxyl radicals and sequester reactive oxygen species), cellular response to oxidative stress, DNA repair, cycle regulation cellular, membrane stabilization, inhibition of the enzyme nicotinamide adenine dinucleotide phosphate oxidase (NADPH-oxidase) and apoptosis. The zinc deficiency, doubled by the high-fat diet, is blamed for the occurrence of cardiac hypertrophy related to obesity. Regarding the role of enzyme cofactor, it influences various structures such as SOD or zinc-α2-glycoprotein (ZAG). The latter is an adipokine with an anti-inflammatory role and in the mobilization of lipids found in reduced quantities in obese patients (129–133). The level of zinc must therefore be kept within normal limits, its marginal deficit may increase the risk of obesity (134). At the same time, Mendes Garrido Abregú F. et al. discuss the importance of adequate zinc levels during breastfeeding. They emphasize that adequate post-weaning zinc diet has a reduced role in preventing cardio-metabolic changes induced by perinatal restriction (135).

Selenium supplementation had, similar to zinc, a beneficial effect in regulating body weight, metabolic and oxidative profile (136, 137). In addition to its antioxidant (main cofactor of GPx) and anti-inflammatory implications, selenium intervenes beneficially in non-alcoholic fatty liver disease induced by obesity. It promotes the synthesis of selenoprotein P1 (SEPP1) which further regulates the Kelch-like ECH-associated protein 1 (KEAP1)/Nrf2 pathway (138, 139). The advantages of exposure to selenium were also documented by Abo El-Magd NF. et al. (140). Last but not least, manganese influences the body’s antioxidant balance. It is a redox-active component with a key role in cellular adaptation to oxidative stress. The main functions are cofactor for SOD and substrate for the formation of manganese non-proteinaceous antioxidants. In these processes, the “adversary” is the iron (141, 142).

#### 3.2.3 Other compounds with an antioxidant role

Flavonoids are plant compounds found in fruits and vegetables, known for their strong antioxidant properties. They are estimated to have a diversity that includes six subgroups made up of up to 5000 substances with a common characteristic (skeletal structures with 15 carbon atoms, two phenyl rings and a heterocyclic ring). The most abundant flavonols are quercetin, catechins and kaempferol. They can be used in the prevention or management of obesity and associated diabetes. It exerts its functions on peripheral tissues and pancreatic beta cells, improving insulin secretion and sensitivity. It also regulates inflammation by acting on NF-κB through mitogen-activated protein kinase (MAPK) pathways. Quercetin can inhibit macrophage inflammatory response by activating AMPK phosphorylation and sirtuin 1 (SIRT1) expression (143–145). Besides these, flavonoids modulate thermogenesis, lipogenesis (decrease), lipolysis (increase), energy consumption, food and nutritional intake, B-oxidation of fatty acids and carbohydrate balance. In the oxidizing balance, flavonoids can clean reactive species of oxygen or nitrogen by direct or indirect mechanism (146–148). A special role is attributed to the ability to stabilize the intestinal microbial ecosystem. Thus, we previously demonstrated that intestinal dysbiosis is a process that can be the basis of a wide range of diseases starting from obesity, the associated comorbidities and culminating in gastrointestinal, cardiovascular, renal, atopy or autoimmunity pathologies. The link between them is attributed to the intestines-vital organs axes, intensively studied at the present time (149–156). Finally, Gentile D. et al. underlines the importance of a diet rich in flavonoids in modulating the systemic inflammatory and oxidative balance, with the aim of avoiding obesity and its associated comorbidities (157).

Alpha-lipoic acid (ALA) is another vegetable component with antioxidant effects. Although it is still under study, research on adults attests that supplementation with 600 mg intravenously for 2 weeks improves insulin sensitivity and reduces mitochondrial functional manifestations. Also, the levels of free fatty acids, dyslipidemia and oxidative/inflammatory markers were reduced, in parallel with adiponectin, which registered an increase. To these is added the positive impact in the recycling of vitamins C and E and the chelation of toxic metals (158–161). Research on murine models is more advanced, indicating similar results. Looking specifically at inflammation and oxidative stress, ALA caused reductions in prostaglandin E2, leukotrienes B4 and C4, T cell proliferation and IL-2 production, and levels of lipid peroxidation products, doubled by increased SOD2 and reduced intracellular glutathione (161–164).

Coenzyme Q10 (ubiquinol/ubiquinone) is a key component in the mitochondrial electron transport chain. One of the causes of ubiquinone deficiency is attributed to the statin-induced lipid-lowering effect by inhibiting the conversion of 3-hydroxy-3-methyl-glutaryl-coenzyme A reductase (HMG-CoA) to mevalonate. The main antioxidant mechanisms attributed to coenzyme Q10 are its action as a cofactor and activator of mitochondrial uncoupling proteins, the ability to accept and donate electrons, the inhibition of lipid and protein peroxidation, the prevention of LDL oxidation and the improvement of the availability of other antioxidants (vitamin C, E, beta-carotene). Thus, a diet with foods rich in coenzyme Q10 (100-150 mg/day) or its supplementation has proven effective in regulating carbohydrate metabolism, improving inflammation and oxidative stress characteristic of obesity/metabolic syndrome (165–168).

The benefits of N-acetyl cysteine (NAC) are multiple and beyond doubt. NAC acts against the complications induced by obesity both by reducing the abnormal pro-inflammatory response and limiting oxidative damage, as well as by inhibiting lipid accumulation by targeting adipogenic transcription factors. Studies on murine models have also demonstrated an effective effect on hyperglycemia, dyslipidemia and oxidative stress (increase the expression of endogenous antioxidant enzymes) occurring consecutively to a diet rich in sucrose (169–172).

The roles of melatonin are vast and still incompletely elucidated. Besides the well-known implications of melatonin in dictating the circadian rhythm, the balance of the intestinal microbiota, sleep disorders and the opioidergic system, it has recently come to the attention of research as a regulatory factor (modulating the activity of melatonin 1 and 2 membrane receptors) of the lipid and tension profile, of glucose metabolism, oxidative stress, inflammation and adipose tissue. Thus, melatonin supplementation between 1-20 mg/day demonstrates a beneficial role in pediatric obesity by reducing associated mitochondrial damage, regulating glycemic homeostasis and increasing the volume/activity of brown adipose tissue. Also, no serious adverse effects were recorded (173–176). At the same time, the use of melatonin as an antioxidant has demonstrated its effectiveness in reducing muscle damage after physical activity in overweight people (177).

Although research on taurine (2-aminoethanesulfonic acid) is limited, its implications in modulating oxidative/metabolic stress, inflammation, insulin sensitivity and vascular remodeling cannot be denied. Thus, its balance can influence the appearance of the affections as well as the predisposition towards the development of associated comorbidities. For estimation purposes, the current scientific literature considers taurine excretion/24 hours inversely correlated with BMI, blood pressure profile and cholesterolemia (178–181). Also, taurine supplementation (3 g/day) doubled by sustained physical exercises, with/without nutritional counseling, demonstrated benefits in reducing inflammation, oxidative stress, preventing endothelial dysfunction induced by a high-fat diet and improving the plasticity of subcutaneous adipose tissue (182–184).

The oral and intestinal microbiota also represent a broad field of research. As I mentioned, its disturbances can be found as risk factors in various pathologies. In particular, at the local level, intestinal dysbiosis can be involved in the pathogenesis of irritable bowel, pancreatitis and celiac disease. It thus achieves a pathogenic interrelation of autoimmunities (see systemic lupus erythematosus). Therefore, the current literature states that the dietary modulation of the intestinal microbiota as well as the transplantation of fecal matter can represent prophylactic and curative strategies in childhood obesity (185–189). Its evaluation currently represents a pinnacle of research. It is known that it is in permanent change since the perinatal period, being influenced by both maternal and individual factors. The first three years are the most important for its optimal definition, the disruptive factors being cesarean birth, artificial feeding, antibiotic therapy (190, 191). Consequently, due to the multiple causal associations with obesity, it is necessary to emphasize the possible beneficial implications of prebiotics/probiotics or symbiotics in its therapeutic modulation. The ways in which they interact with the homeostasis of the internal environment playing the role of antioxidants are vast, from the clearance of reactive substances to the stimulation of signaling with the increase of the cytoprotective capacity of the host (192, 193). They have proven benefits in the co-adjuvant therapy of obesity, insulin resistance, diabetes and non-alcoholic fatty liver disease (194). The observation was demonstrated in studies on adult populations, when supplemented with *Saccharomyces boulardii* and SOD for two months. In addition, the increase in the level of vitamin D was noted (195).

**Table 1** illustrates the main means of action of enzymatic and non-enzymatic antioxidants in mitigating the oxidative stress present in obesity.

**Table 1:** Means to combat oxidative stress in pediatric obesity.

The type of antioxidant	Mode of action
Enzymatic antioxidants	
*Superoxide dismutase*	- the first line of defense against reactive oxygen species, converting superoxide radicals into hydrogen peroxide
*Catalase*	- degrades hydrogen peroxide into oxygen and water
*Glutathione peroxidase*	- modulates the balance between necessary and harmful levels of reactive oxygen species by regulating the accumulation of hydrogen peroxide - is the main supplier of reduced glutathione, a necessary substrate for the proper development of the redox process - has a neuroprotective, anti-inflammatory effect, regulates metabolism and cardiovascular homeostasis, being also a valuable anti-oncogenic factor - its overexpression can have negative consequences
*Peroxiredoxin*	- catalyze the reduction of peroxides (e.g., hydrogen peroxide, organic peroxides) to water and alcohol, using thiols (e.g., thioredoxin or glutathione) as electron donors
Non-enzymatic antioxidants	
*Vitamin A*	- neutralizes free radicals, stabilizing them and preventing chain reactions that lead to cell damage - protects the constituent lipids of cell membranes against lipid peroxidation - modulates gene expression by binding to specific nuclear receptors, regulating the expression of genes that encode antioxidant enzymes - potentiates the effects of other antioxidants through synergy
*Vitamin E*	- similar to vitamin A
*Vitamin C*	- neutralizes free radicals through the effect of donating an electron, converting them into less reactive molecules - contributes to the regeneration of other antioxidants (e.g. vitamin E) - participates in the synthesis of collagen, an important structural and functional protein in the skin, blood vessels, bones and other connective tissues - confers protection to DNA and proteins against oxidative damage, helping to maintain the genetic and functional integrity of cells - modulates the expression of genes involved in antioxidant and anti-inflammatory responses - improves the absorption of non-heme iron from plant-based foods, helping to maintain adequate iron levels in the body
*Vitamin D*	- modulates through the receptor the expression of genes involved in antioxidant defense (e.g., glutathione peroxidase, superoxide dismutase, catalase) - has anti-inflammatory properties, reducing the production of pro-inflammatory cytokines (e.g., TNF-α and IL-6) - potentiates the synthesis of glutathione, one of the most important intracellular antioxidants prevents lipid peroxidation
*Zinc*	- contributes to maintaining the structural and functional integrity of cells, preventing lipid peroxidation - acts as a cofactor for antioxidant enzymes (e.g. superoxide dismutase with zinc and copper) inhibiting the production of free radicals - protects against DNA and protein damage modulates the inflammatory response - supports the immune system
*Cooper*	- plays the role of cofactor for antioxidant enzymes participates in the synthesis and functioning of cytochrome c oxidase, thus supporting mitochondrial function - supports the activity of ceruloplasmin, a transport protein with an antioxidant role (prevents the formation of free radicals through Fenton reactions) modulates the immune system - promotes the formation of collagen and elastin
*Iron*	- cofactor for antioxidant enzymes (e.g., catalase, peroxidase) - participate in cellular metabolism and energy production - essential for the functioning of enzymes in the electron transport chain in the mitochondrion - role in the synthesis of hemoglobin and myoglobin, proteins that transport oxygen in the blood and muscles and modulate immune responses - participates in the detoxification of free radicals
*Selenium*	- component of antioxidant enzymes neutralizes free radicals - participate in the regeneration of other antioxidants (e.g. vitamin C, vitamin E) - supports the immune system - *adequate levels of selenium are associated with improved immune function and increased ability to fight infections* - and reduces inflammation
*Chromium*	- regulates glucose and insulin levels - reduces inflammation associated with insulin resistance - supports mitochondrial function - can contribute to the activation of some antioxidant enzymes (e.g. superoxide dismutase, glutathione peroxidase)
*Magnesium*	- neutralizes free radicals and prevents oxidative damage at the cellular level - can inhibit oxidative reactions and decrease free radical levels in cells. - is involved in the activation of essential antioxidant enzymes (e.g. superoxide dismutase and glutathione peroxidase) - supports mitochondrial function - regulates inflammation - protects DNA and proteins against oxidative damage - reduces the risk of chronic diseases
*Manganese*	- essential cofactor for manganese superoxide dismutase - protects cell membranes against oxidative damage through the ability to neutralize free radicals that can attack lipids in cell membranes, thus preventing lipid peroxidation - is involved in the functioning of other antioxidant enzymes (e.g., glutathione peroxidase, catalase), helping to detoxify hydrogen peroxide and other reactive oxygen species - role in glucose and lipid metabolism - supports the immune system
*Flavonoids*	- reduces free radical levels - it has anti-inflammatory properties and improves insulin sensitivity - they intervene in reducing fat accumulation in the body and improving lipid metabolism - intervene in the protection and regeneration of mitochondrial function
*α-lipoic acid*	- neutralizes a variety of free radicals and reactive oxygen species intervenes in the regeneration of other antioxidants (e.g. vitamin C, vitamin E, glutathione) - reduce inflammation - improves insulin resistance - protect mitochondria against oxidative damage and dysfunction
*Coenzyme Q10*	- neutralizes free radicals and reactive oxygen species - helps protect cell membranes against lipid peroxidation - is essential for the optimal functioning of mitochondria, helping to reduce the excessive production of free radicals and to prevent oxidative stress associated with mitochondrial dysfunctions - improves endothelial dysfunction - can increase the activity of other antioxidant enzymes (e.g. superoxide dismutase, glutathione peroxidase)
*Melatonin*	- lowers free radical levels - protects mitochondria against oxidative damage and improves their function - reduce inflammation - improves the antioxidant function of other enzymes - regulates lipid and carbohydrate metabolism, contributing to the reduction of fat accumulation and the prevention of metabolic dysfunctions associated with obesity
*Taurine*	- neutralizes free radicals and reduces oxidative stress - protects the liver against oxidative stress, thus reducing the risk of liver damage associated with obesity - reduce inflammation - improves insulin sensitivity - it protects the kidneys against oxidative stress and may contribute to the maintenance of renal homeostasis

## 4 The role of diet in obesity management

Continuing the appropriate screening of the population at risk, both the targeted therapeutic approach (pharmacological or surgical) and the adjunctive management of pediatric obesity (sleep hygiene, balanced diet, avoiding sedentary lifestyle, sustained physical exercise) is a broad topic of ongoing research. According to the current consensus, multidisciplinary management aims to prevent and counteract the underlying pathology and associated comorbidities, efforts aimed at maintaining the psychosocial integrity of the child (196–198).

In practical terms, international guidelines currently support the approach of a minimum of 60 minutes/day of moderate to vigorous physical exercises. The most effective method to eliminate excess weight and reduce waist circumference has proven to be the combination of aerobics and resistance training. An important factor in this approach is the school. Studies have shown that encouraging physical activity in schools has led to improvements in BMI, waist circumference in women, skinfold thickness and body fat (197, 199). Undoubtedly, in order to be effective, physical activity must be a part of changing the entire lifestyle. Thus, it was proven that the lack of breakfast, the frequent consumption of snacks, the increase in the amount of fat and carbohydrates in the diet and a narrow variability of healthy food options (e.g., fruits, vegetables and dairy) were precipitating factors of childhood obesity. Added to these are the consumption of sugar-sweetened beverages or fast food (199–201).

The pharmacological intervention possibilities are more restrictive compared to those in the adult population. The main consideration resides in the lack of substance certification studies. Therefore, the pharmacological substances currently approved for obesity are Orlestin, glucagon-like peptide-1 analogues, Liraglutide, Setmelanotide and Metreleptin. The means of action, dosage and indications are varied, thereby ensuring the possibility of an individualized therapy. We also note Semaglutide and Exenatide as therapeutic options in research regarding the utility in obesity (199).

Bariatric surgery represents a therapeutic alternative dedicated to severe cases of obesity, refractory to the previously described hygienic-dietary and pharmacological measures and which associate comorbidities. Its indications are defined depending on the value of the body mass index. We thus recognize the usefulness of bariatric surgery when BMI >40 kg/m2 or BMI >35 kg/m2 with significant comorbidities. The possible surgical techniques are quite varied, and BMI reduction seems to be effective in a follow-up interval of 1-5 years post-procedural. Additionally, this has proven to be beneficial in improving associated comorbidities (e.g., diabetes, hypertension, dyslipidemia, and proteinuria). The studies that will unequivocally certify the role of bariatric surgery and the superiority of each technique are currently underway (199, 201). One such example is a multicenter study undertaken by Järvholm K. et al. (202).

Finally, in accordance with what was stated previously, Stabouli S. et al. support the need for future research on the early detection of risk factors and the elucidation of the mechanisms underlying pediatric obesity (203). Of these, we detailed throughout the manuscript the pathophysiology implications of antioxidant substances in the evolutionary pattern of obesity. Therefore, in order to add practical utility to the work, **Table 2** refers to the main foods with a high content of antioxidants, beneficial in the diet of children and obese adolescents.

**Table 2:** Food sources rich in antioxidants (adapted from Webster-Gandy J. et al., Kozłowska A. et al., Alam MA. et al., Meng X. et al., Wu G. et al. and Shay KP. et al.) (165, 204–208).

Antioxidants	Food
Vitamins A	• Liver and liver products; • Kidney and offal; • Oily fish and fish liver oils; • Eggs; • Carrots; • Red peppers; • Spinach; • Broccoli; • Tomatoes.
E	• Wheat germ oil; • Almonds; • Sunflower seeds & oil; • Safflower oil; • Hazelnuts; • Peanuts & peanut butter; • Corn oil.
C	• Kiwi fruit; • Citrus fruit (oranges, lemons, satsumas, clementines, etc.); • Black currants; • Guava; • Mango; • Papaya; • Pepper; • Brussels sprouts; • Broccoli; • Sweet potato.
D	• Cod liver oil; • Oily fish (salmon, mackerel, etc.); • Milk; • Margarine; • Breakfast cereals; • Eggs; • Liver.
Microelements Zinc	• Lamb; • Leafy & root vegetables; • Crabs & shellfish; • Beef; • Offal; • Whole grains; • Pork; • Poultry; • Milk and milk products; • Eggs; • Nuts.
Copper	• Offal; • Nuts; • Cereals & cereal products; • Meat & meat products.
Iron	• Meat especially offal; • Fish; • Eggs; • Meat extracts; • Bread & flour; • Breakfast cereals; • Vegetables (dark green) & pulses; • Nuts & dried fruit—prunes, figs, apricots; • Yeast extract.
Magnesium	• Green vegetables; • Pulses & whole grain cereals; • Meats.
Selenium	• Offal; • Fish; • Brazil nuts; • Eggs; • Poultry; • Meat and meat products.
Chromium	• Meat; • Whole grains; • Legumes; • Nuts.
Manganese	• Cereals & cereal products; • Tea; • Vegetables.
Other antioxidants Flavonoids	• Fruits; • Vegetables; • Nuts; • Seeds; • Spices.
α-lipoic acid	• Muscle meats; • Heart; • Kidney; • Liver.
Coenzyme Q10	• Meat; • Poultry; • Fish.
Melatonin	• Eggs; • Over; • Cereals (corn, rice); • Fruits (grapes, strawberries); • Vegetables (tomatoes, peppers, mushrooms); • Seeds (white and black mustard, soy, flax, beans); • Nuts (pistachio); • Coffee; • Balsamic vinegar; • Extra virgin olive oil.
Taurine	• Beef.

## 5 Conclusions

Obesity is found both in childhood/adolescence and in adulthood. Many pathological processes compete with its induction and maintenance. Therefore, the unbalanced lifestyle is only the tip of the iceberg. The current work achieved its goal of bringing the concept of pediatric obesity and accompanying comorbidities up to date. Most of the obese children, without therapeutic interventions, will evolve further into obese adults that can also associate metabolic syndrome. In order to improve/stop the systemic decline, measures aimed at lifestyle modification, pharmacotherapy or surgical therapy are considered. Knowing the impact played by released free radicals as a consequence of oxidative stress, we discussed the main endogenous or exogenous antioxidant substances that interfere with the pathological process, briefly detailing the roles of each. Although the implications of antioxidants in childhood obesity are certified by specialized medical literature, we identified in our search a reporting bias of the main food sources rich in substances with an antioxidant role. Consequently, we chose to do a brief review of them. In conclusion, the current study places the obesity-oxidative stress-antioxidants relationship in a different light. We consider it opportune to widen the horizons in this direction by designing and implementing screening programs and individualized supplementation of nutritional deficiencies in antioxidant substances with the aim of reducing the incidence and burden of pediatric obesity.

## Author contributions

AL: Conceptualization, Investigation, Writing – original draft. SF: Investigation, Methodology, Writing – original draft. EJ: Investigation, Methodology, Writing – original draft. IS: Investigation, Software, Writing – original draft. II: Investigation, Software, Writing – original draft. AK: Validation, Visualization, Writing – review & editing. DS: Software, Validation, Writing – review & editing. MS: Validation, Visualization, Writing – review & editing. OC: Investigation, Software, Writing – original draft. NR: Supervision, Validation, Writing – review & editing. CMM: Writing – review & editing, Visualization. VL: Methodology, Supervision, Writing – review & editing. AN: Project administration, Validation, Writing – review & editing.
