# Corrigendum: Is oxidative stress - antioxidants imbalance the physiopathogenic core in pediatric obesity?

**DOI:** 10.3389/fimmu.2025.1555244

**Published:** 2025-01-13

**Authors:** Ancuta Lupu, Silvia Fotea, Elena Jechel, Iuliana Magdalena Starcea, Ileana Ioniuc, Anton Knieling, Delia Lidia Salaru, Maria Oana Sasaran, Olga Cirstea, Ninel Revenco, Cristina Maria Mihai, Vasile Valeriu Lupu, Alin Horatiu Nedelcu

**Affiliations:** ^1^ Pediatrics, “Grigore T. Popa” University of Medicine and Pharmacy, Iasi, Romania; ^2^ Clinical Medical Department, Faculty of Medicine and Pharmacy, “Dunarea de Jos” University, Galati, Romania; ^3^ Faculty of Medicine, “Grigore T. Popa” University of Medicine and Pharmacy, Iasi, Romania; ^4^ Pediatrics, “George Emil Palade” University of Medicine, Pharmacy, Science and Technology, Targu Mures, Romania; ^5^ Pediatrics, Nicolae Testemitanu State University of Medicine and Pharmacy, Chisinau, Moldova; ^6^ Pediatrics, Faculty of Medicine, “Ovidius” University, Constanta, Romania

**Keywords:** oxidative stress, endogenous antioxidant systems, exogenous antioxidants, obesity, diet, immunity, child

In the published article, an author name was incorrectly written as “Neli Revenco”. The correct spelling is “Ninel Revenco”.

The authors apologize for this error and state that this does not change the scientific conclusions of the article in any way. The original article has been updated.

